# Biosynthesis, Chemical Structure, and Structure-Activity Relationship of Orfamide Lipopeptides Produced by *Pseudomonas protegens* and Related Species

**DOI:** 10.3389/fmicb.2016.00382

**Published:** 2016-03-30

**Authors:** Zongwang Ma, Niels Geudens, Nam P. Kieu, Davy Sinnaeve, Marc Ongena, José C. Martins, Monica Höfte

**Affiliations:** ^1^Laboratory of Phytopathology, Crop Protection, Faculty of Bioscience Engineering, Ghent UniversityGhent, Belgium; ^2^NMR and Structure Analysis Unit, Department of Organic and Macromolecular Chemistry, Ghent UniversityGhent, Belgium; ^3^Microbial Processes and Interactions Unit, Faculty of Gembloux Agro-Bio Tech, University of LiègeGembloux, Belgium

**Keywords:** fluorescent pseudomonads, nonribosomal peptide synthase, orfamides, biocontrol, *Magnaporthe oryzae*, *P. protegens* CHA0 and Pf-5

## Abstract

Orfamide-type cyclic lipopeptides (CLPs) are biosurfactants produced by *Pseudomonas* and involved in lysis of oomycete zoospores, biocontrol of *Rhizoctonia* and insecticidal activity against aphids. In this study, we compared the biosynthesis, structural diversity, *in vitro* and *in planta* activities of orfamides produced by rhizosphere-derived *Pseudomonas protegens* and related *Pseudomonas* species. Genetic characterization together with chemical identification revealed that the main orfamide compound produced by the *P*. *protegens* group is orfamide A, while the related strains *Pseudomonas* sp. CMR5c and CMR12a produce orfamide B. Comparison of orfamide fingerprints led to the discovery of two new orfamide homologs (orfamide F and orfamide G) in *Pseudomonas* sp. CMR5c. The structures of these two CLPs were determined by nuclear magnetic resonance (NMR) and mass spectrometry (MS) analysis. Mutagenesis and complementation showed that orfamides determine the swarming motility of parental *Pseudomonas* sp. strain CMR5c and their production was regulated by *luxR* type regulators. Orfamide A and orfamide B differ only in the identity of a single amino acid, while orfamide B and orfamide G share the same amino acid sequence but differ in length of the fatty acid part. The biological activities of orfamide A, orfamide B, and orfamide G were compared in further bioassays. The three compounds were equally active against *Magnaporthe oryzae* on rice, against *Rhizoctonia solani* AG 4-HGI in *in vitro* assays, and caused zoospore lysis of *Phytophthora* and *Pythium*. Furthermore, we could show that orfamides decrease blast severity in rice plants by blocking appressorium formation in *M. oryzae*. Taken all together, our study shows that orfamides produced by *P. protegens* and related species have potential in biological control of a broad spectrum of fungal plant pathogens.

## Introduction

The majority of natural rhizosphere soil microorganisms in various ecosystems is composed of Proteobacteria (Philippot et al., 2013). Within this group rhizosphere-derived fluorescent pseudomonads have received a lot of attention as biocontrol agents in the past few decades (Höfte and Altier, 2010; Olorunleke et al., 2015a). Rhizosphere-derived fluorescent pseudomonads have the ability to swiftly adapt to changeable environmental conditions and efficiently colonize the plant root system, and play critical roles in the interspecies interaction among plants, plant pathogens, bacterial predators, and other biotic and abiotic stresses from natural environments. *Pseudomonas* species show an enormous metabolic versatility and some isolates produce a remarkable spectrum of secondary metabolites both *in vitro* and *in vivo* conditions. These secondary metabolites are indispensably involved in interspecies interactions in the soil environment (Raaijmakers and Mazzola, 2012).

Various *Pseudomonas* biocontrol strains produce CLP type biosurfactants (Olorunleke et al., 2015a). CLPs are amphiphilic molecules composed of a cyclic oligopeptide lactone ring coupled to a fatty acid tail (Raaijmakers et al., 2006, 2010). These molecules are synthesized by non-ribosomal peptide synthases (NRPSs), which normally encode different modules containing several domains for loading, selecting, synthesizing amino acids and after their configuration, the peptide is finally released by thiolation domain(s) (Strieker et al., 2010). CLPs possess broad spectra activities, such as antibiosis against bacteria, fungi, protozoa, and human tumor cell lines (Raaijmakers et al., 2010; Roongsawang et al., 2010) and have potential as pharmaceutical candidates or for the biocontrol of plant pathogens (Banat et al., 2010, 2014; D'aes et al., 2010; Sachdev and Cameotra, 2013). *Pseudomonas* derived CLPs are currently divided in eight different structural groups that differ in length and composition of the oligopeptide and fatty acid tail (Olorunleke et al., 2015a).

Orfamides were first discovered in the well-studied biocontrol strain *Pseudomonas protegens* Pf-5 by a novel genomisotopic approach, and the chemical structure of orfamide A was determined by NMR and MS analysis (Gross et al., 2007). Orfamides consist of 10 amino acids and a 3-hydroxydodecanoic or tetradecanoic acid tail. Genome mining and chemical analysis revealed that *Pseudomonas* sp. CMR12a, a biocontrol strain obtained from cocoyam roots in Cameroon, can produce orfamide B and the new homologs orfamide D and E (D'aes et al., 2014). *Pseudomonas* sp. CMR12a is related to *P. protegens*, but phylogenetically clearly distinct (Ruffner et al., 2015). Orfamides are important determinants for bacterial surface motility and show surface tension reduction activity (Gross et al., 2007; Jang et al., 2013; D'aes et al., 2014). In addition, orfamide A produced by *P*. *protegens* F6 showed dose-dependent insecticidal activity against aphids in greenhouse biocontrol trails (Jang et al., 2013). Orfamide B, produced by *Pseudomonas* sp. CMR12a, can affect the hyphal growth of *Rhizoctonia solani*, and soil assays with orfamide biosynthesis mutants revealed that orfamides work together with phenazine antibiotics or sessilin-type CLPs in the biocontrol activity against root rot caused by *R. solani* in bean plants (Olorunleke et al., 2015b).

*Pseudomonas* sp. CMR5c is a biocontrol strain from cocoyam roots in Cameroon related to *Pseudomonas* sp. CMR12a (Perneel et al., 2007). This strain produces multiple antibiotics such as phenazines, pyrrolnitrin and pyoluteorin, and also has biosurfactant activity (Perneel et al., 2007). Recently, the genome of *Pseudomonas* sp. CMR5c was sequenced, revealing the presence of diacetylphloroglucinol biosynthesis genes, the Fit gene cluster that contributes to insecticidal activity and an NRPS cluster involved in CLP biosynthesis (Flury et al., 2016). In this work, we identified the NRPS genes and CLPs produced by *Pseudomonas* sp. CMR5c. In addition, we performed a detailed analysis and comparison of genetic biosynthesis, phylogeny, and chemical structures of orfamides produced by *Pseudomonas* sp. CMR5C, *P. protegens* and related species. Using three structurally different orfamides, we tested the hypothesis that length of the fatty acid tail and/or amino acid substitutions in the peptide moiety may affect biological activity. Our study revealed that all three compounds are equally active against several plant pathogenic fungi and oomycetes and inhibit appressoria formation in the rice blast fungus *Magnaporthe oryzae*.

## Materials and methods

### Strains, media, and growth conditions

Bacteria, fungal strains, and plasmids used in this study are shown in Table 1. *Pseudomonas* strains were routinely maintained on Luria-Bertani (LB) medium (Sambrook et al., 1989) at 28°C. *E. coli* WM3064 was cultured on LB medium contained 100 μg/mL of 2,6-diaminopimelic acid (DAP) at 37°C (Saltikov and Newman, 2003). Gentamicin was added to LB medium at 25 μg/mL for *E. coli* WM3064 and at 100 μg/mL for *Pseudomonas* strains, respectively. *Saccharomyes cerevisiae* InvSc was grown on yeast peptone dextrose medium at 30°C. Solid medium and soft agar medium contained 1.5% (*w*/*v*) and 0.6% (*w*/*v*) agar, respectively. Liquid cultures of *Pseudomonas* strains were obtained in liquid King'B (KB) (King et al., 1954) medium on a rotary shaker with stirring rate of 150 rpm. *M. oryzae* isolate VT5M1 (Thuan et al., 2006) was cultured on complete medium (CM) (Talbot et al., 1993). *Phytophthora porri* isolate CBS 127099 was maintained on V8 agar (Bertier et al., 2013). *R. solani* AG 4-HGI isolate CuLT-Rs36 (Nerey et al., 2010) and *Pythium ultimum* were routinely cultivated on potato dextrose agar (PDA; Difco).

**Table 1:** **Strains, reference sequences, and plasmids used in this study^a^**.

**Strain or plasmid**	**Relevant characteristics**	**References or source**
***Pseudomonas*** **STRAINS**		
*P. protegens* CHA0	Orfamide^+^, derived from rhizosphere of tobacco, Switzerland	Stutz et al., 1986
*P. protegens* Pf-5	Orfamide^+^, derived from rhizosphere of cotton, Texas, USA	Howell and Stipanovic, 1979
CMR12a	Orfamide^+^, sessilin^+^, wild type derived from rhizosphere of red cocoyam, Cameroon	Perneel et al., 2007
CMR12a-Clp1	Orfamide^+^, sessilin^−^; mutant with insertion in sessilin (CLP1) biosynthesis genes, Gm^R^	D'aes et al., 2011
CMR5c	Orfamide^+^, wild type derived from rhizosphere of red cocoyam, Cameroon	Perneel et al., 2007
CMR5cΔ*ofa*	Orfamide^−^, mutant with deletion in *ofaB* and *ofaC* genes	This study
CMR5cΔ*luxRup*	Orfamide^−^, mutant with deletion in *luxR* type transcriptional regulator gene located upstream of orfamide biosynthetic genes	This study
CMR5cΔ*luxRdown*	Orfamide^−^, mutant with deletion in *luxR* type transcriptional regulator gene located downstream of orfamide biosynthetic genes	This study
***Pseudomonas*** **SEQUENCES**		
*P*. *protegens* Cab57	Orfamide^+^, derived from rhizosphere of shepherd's purse, Japan	Takeuchi et al., 2014
PH1b	Derived from the phytotelma of a carnivorous plant, Malaysia	NCBI database
CMAA1215	Derived from Brazilian mangroves, Brazil	Vasconcellos et al., 2013
Wayne1R	Biological control strain derived from rhizosphere of corn, Wayne, Pennsylvania, USA	Rong et al., 2012
*Saccharomyes cerevisiae* InvSc1	Yeast strain for *in vivo* recombination (*ura3-52/ura3-52* mutation)	Invitrogen
*E. coli* WM3064	Donor strain for conjugation; DAP^−^	Saltikov and Newman, 2003
**PLASMID**		
PMQ30	Gene replacement vector for *Pseudomonas* species; *sacB*, URA3, Gm^R^	Shanks et al., 2006
PMQ30-Δ*ofa*	Vector for site specific mutagenesis of orfamide biosynthesis genes	This study
PMQ30-Δ*luxRup*	Vector for site specific mutagenesis of *luxRup* biosynthesis gene	This study
PMQ30-Δ*luxRdown*	Vector for site specific mutagenesis of *luxRdown* biosynthesis gene	This study
*Magnaporthe oryzae* isolate VT5M1	Causal agent of rice blast disease	Thuan et al., 2006
*Rhizoctonia solani* AG 4-HGI CuLT-Rs36	Causal agent of root rot disease on bean	Nerey et al., 2010
*Phytophthora porri* CBS 127099	Oomycete pathogen causing white tip disease on leek	Bertier et al., 2013
*Pythium ultimum*	Oomycete pathogen causing root rot on cucumber	Lab stock

a *Gm^R^, gentamicin resistance; DAP, 2, 6-diaminopimelic acid; DAP^−^, DAP auxotroph; CLP1, sessilin type lipopeptide*.

### Genome mining and bioinformatic analyses

The genomic sequences of selected *Pseudomonas* strains (Table 1) were retrieved from Genbank database and submitted to RAST server (Aziz et al., 2008) and antiSMASH 3.0 (Weber et al., 2015) for automated genome annotation and genome mining. Alternatively, genome mining of NRPS biosynthetic gene clusters was conducted by BLAST search (http://blast.ncbi.nlm.nih.gov/Blast.cgi) and through comparison with reported sequences. Gaps of scaffold sequences of *Pseudomonas* sp. CMR5c were filled by polymerase chain reaction (PCR) and sequencing (LGC Genomics). Domains of NRPS gene clusters were obtained by using NRPSpredictor2 (Röttig et al., 2011) and NRPS-PKS program (Ansari et al., 2004). Similarity of selected proteins was identified by BLAST search (http://blast.ncbi.nlm.nih.gov/Blast.cgi). Phylogeny trees were constructed with Molecular Evolutionary Genetics Analysis version 5 (MEGA5) (Tamura et al., 2011). The draft genome of *Pseudomonas* sp. CMR5c has recently been released (Flury et al., 2016). Genomic sequences of *Pseudomonas* sp. CMR5c were deposited in GenBank database under accession number: NZ_LHUY01000001—NZ_LHUY01000044 (Flury et al., 2016). The biosynthetic gene cluster of orfamide identified from genomic sequences of *Pseudomonas* sp. CMR5c was deposited in GenBank database under accession number KT613918. Selected *Pseudomonas* strains used in this study are shown in Table 1 and their genomic sequences are publicly available in GenBank. The full sequences of *rpoD* and *gyrB* of selected *Pseudomonas* strains were directly retrieved from GenBank database and used for further phylogeny analysis in this study.

### Swarming motility and droplet collapse assays

Overnight cultures of *Pseudomonas* strains were maintained in liquid LB medium, and washed two times with sterilized deionized water. Bacterial concentration was adjusted to optical density of 0.9 (620 nm). Soft agar plates were prepared by adding 0.6% (*w*/*v*) agar in LB medium. Five microliters of aliquots were carefully spotted in the center of soft agar plates, and plates were incubated at 28°C. All strains were tested in triplicates. Swarming was observed 20 h after incubation. Droplet collapse assay was conducted exactly the same as reported previously (Gross et al., 2007).

### Site directed mutagenesis

Site directed deletion of targeted genes of *Pseudomonas* sp. CMR5c was conducted by allelic replacement using plasmid PMQ30 as reported before (Shanks et al., 2006; D'aes et al., 2014). Primer pairs used for generation of deletion mutants are shown in Supplementary Table 1. DNA fragments upstream (Up fragment, Supplementary Table 1) and downstream (Down fragment, Supplementary Table 1) of the genes of interest, were amplified by PCR using corresponding primer pairs and detected by 2% (*w*/*v*) agarose gel electrophoresis. These two fragments were subsequently cloned next to each other via *in vivo* recombination in the yeast *S. cerevisiae* InvSc1. The resulting deletion plasmids PMQ30-Δ*ofa*, PMQ30-Δ*luxRup*, and PMQ30-Δ*luxRdown* were isolated from *S*. *cerevisiae* InvSc by GeneJET Plasmid Miniprep Kit (Thermo Scientific) and introduced into *E*. *coli* WM3064 by electroporation. Recombination in the plasmid was confirmed by sequencing (LGC Genomics). The plasmids were mobilized into *Pseudomonas* sp. CMR5c by conjugation with *E*. *coli* WM3064. *Pseudomonas* strains containing the plasmid were selected on LB medium contained 10% (*w*/*v*) sucrose without sodium chloride. Deletion mutants that grew on LB with 10% (*w*/*v*) sucrose but did not grow on LB with 100 μg/mL gentamicin were selected and the deletion was confirmed by PCR. Ultrahigh performance liquid chromatography-mass spectrometry (UPLC-MS) was used for chemical characterization of deletion mutants. Phenotypic characterization of deletion mutants was conducted by swarming motility assay on soft agar plates.

### MS and NMR analysis

MS analysis of samples from cultures of pseudomonads and purified compounds were conducted on a reversed-phase UPLC-MS system, consisting of an UPLC (Waters, Acquity class H) coupled with a single quadrupole detector (Waters, Acquity) on an Acquity UPLC BEH C18 (ϕ 2.1 × 50 mm, 1.7 μm, Waters); or on a liquid chromatography-mass spectrometry (LC-MS) 1100 Series HPLC system (Agilent Technologies) with a type VL electrospray ionization detector and equipped with a Luna C18 (2) reversed-phase column (ϕ 4.6 × 250 mm, 5 μm; Phenomenex, Torrance, CA, USA). NMR measurements of purified compounds were recorded on a Bruker Avance III spectrometer operating at 500.13 and 125.76 MHz for ^1^H and ^13^C frequencies, respectively. All measurements were performed in CD_3_CN solution and at 298K. One-dimensional (^1^H-NMR, ^13^C-NMR), and two-dimensional NMR spectroscopy, in particular correlation spectroscopy (^1^H-^1^H COSY), total-correlation spectroscopy (^1^H-^1^H TOCSY), rotating-frame nuclear Overhauser effect spectroscopy (^1^H-^1^H ROESY), heteronuclear single-quantum correlation (^1^H-^13^C HSQC) spectroscopy, and heteronuclear multiple-bond correlation (^1^H-^13^C HMBC) spectroscopy was performed for purified compounds.

### Isolation and purification of CLPs

A seed culture of *Pseudomonas* strains was obtained in 250-mL flasks containing 50 mL liquid KB medium placed on a shaker for 24 h at 28°C, and subsequently inoculated into 2-L flasks containing 500 mL liquid KB medium with a stirring rate of 150 rpm for 48 h. The crude extracts of CLPs from *Pseudomonas* cultures were prepared following a protocol published previously (Ma et al., 2012). More specifically, *Pseudomonas* supernatant was collected after centrifugation at 10,000 *g* for 10 min, acidified to pH 2.0 with 6 M hydrochloric acid, and then kept overnight at 4°C. The precipitate was collected after centrifugation at 10,000 *g* for 10 min and extracted with methanol. The organic phase was collected by centrifugation at 10,000 *g* for 10 min and dried without vacuum at room temperature, yielding crude extracts. Crude extracts of CLPs were further separated by gradient acetonitrile/H_2_O (20, 40, 60, 80, 100%, v/v) on a standard C18 SPE cartridge (900 mg, Grace™Alltech™). The fractions containing CLPs were detected by droplet collapse assay on parafilm, and the presence of CLPs was confirmed by UPLC-MS analysis. Fractions containing CLPs were collected, dried, and yielded semi-purified CLPs that were further separated by semi-preparative reversed-phase high performance liquid chromatography (RP-HPLC) on a Luna C-18 (2) (Φ 10 × 250 mm, Phenomenex, Torrance, CA, USA) column by repeated injections (sandwich mode), eluted by 90–100% (v/v) acetonitrile in HPLC-grade water [Trifluoroacetic acid 0.1% (v/v)] over 30 min, with flow rate of 3.5 mL/min and detection wavelength of ultraviolet 214 nm. Orfamide A was purified from *P*. *protegens* CHA0, orfamide B, and G were purified from *Pseudomonas* sp. CMR5c.

Samples of *Pseudomonas* sp. CMR12a for UPLC-MS analysis were prepared from cells on soft agar plate since orfamide secretion in this strain is hampered by the presence of sessilin (D'aes et al., 2014). The cells of *Pseudomonas* sp. CMR12a were collected, suspended in 50% (v/v) acetonitrile/H_2_O solution and ultra-sonicated (Sonoplus, Bandelin Electronic, Berlin) for 1 min. The sample was collected from the supernatant after centrifugation (10,000 *g* for 5 min) of sonicated mixture. Samples of other *Pseudomonas* strains were prepared from supernatant of overnight liquid KB cultures.

### Bioassays with *M. oryzae*

Unless stated otherwise, stocks of orfamides were prepared in pure dimethyl sulfoxide (DMSO) and diluted in water to desired concentrations for further bioassays. Sporulation of *M. oryzae* isolate VT5M1 was obtained following a protocol published previously (Thuan et al., 2006). Spores (5 × 10^4^ per milliliter) of 5-day old *M. oryzae* isolate VT5M1 were collected from CM plates and co-incubated with orfamide solutions, while controls only received the same amount of DMSO. Spore germination was evaluated by counting the number of germination tube of spores after 4 h incubation at 28°C. Appressorium formation was tested by putting a 10-μL spore solution on a glass slide cover (12 × 12 mm) after 8 h incubation in dark condition at 28°C, and the number of appressoria were counted by randomly selecting at least 50 spores. Appressorium formation in planta was assessed as described previously (Koga, 1994). Rice sheaths were inoculated with spore solutions treated or not with orfamides. Representative pictures were taken 24 h post treatment. All microscopic observations were made using an Olympus BX51 microscope. *In vitro* antagonistic assay of orfamides against *M. oryzae* isolate VT5M1 was conducted by paper-agar disc diffusion assay, exactly as described previously (Ma et al., 2012). All experiments were repeated three times.

### Rice biocontrol assay

Rice cultivar *indica* cv. CO-39 was used in plant experiments. Rice seeds were surface sterilized with 1% (*w*/*v*) sodium hypochlorite solution for 5 min and washed five times with deionized water. Surface sterilized seeds were germinated on moistened filter paper in Petri dishes (9 cm diameter) for 5 days, and planted into plastic trays with potting soil (23 × 16 × 6 cm, 12 plants per tray), as described previously (De Vleesschauwer et al., 2006). Rice was routinely maintained in greenhouse conditions with a photoperiod of 12 h light at 30 ± 4°C. Four-week old (five-leaf stage) rice plants were used for further bioassays.

Spores of 5-day old *M. oryzae* isolate VT5M1 were collected from CM plates and suspended into 0.5% (*w*/*v*) gelatin to a final concentration of 5 × 10^4^ per milliliter. Fifty millimolars of purified CLP samples were prepared as stock solution in DMSO, and then diluted to the needed concentration for further bioassays. CLPs and *M. oryzae* spores were mixed thoroughly and evenly sprayed onto rice leaves by a compressor-powered airbrush gun, while control plants were only sprayed with the same amount of DMSO treated spores. Each plant received 1 mL spore solution. Disease score was conducted 6 days post infection, by counting the number of sporulating susceptible-type blast lesions per 10 cm of the second youngest leaves of rice plants, as described previously (De Vleesschauwer et al., 2006). Results are expressed as relative infection values compared to control plants. Pictures of typical disease symptoms were taken 7 days after infection. The biocontrol assay was repeated in time and two trays (=24 plants) were used in each experiment.

### *In vitro* antibiosis assay

Microscopic assays showing the effect of orfamide treatments on hyphal branching of *R. solani* AG 4-HGI were carried out as described by Bolwerk et al. (2003) and Olorunleke et al. (2015b). Zoospores of *P. porri* CBS 127099 and *P. ultimum* were harvested and collected as described earlier (de Bruijn et al., 2007). Zoospores were treated with different concentrations of orfamides and time to zoospore lysis was evaluated microscopically. Experiments were repeated independently three times and the data shown are representative for one observation.

## Results

### *In silico* analysis of orfamide synthases and flanking regions

**Figure 1:**
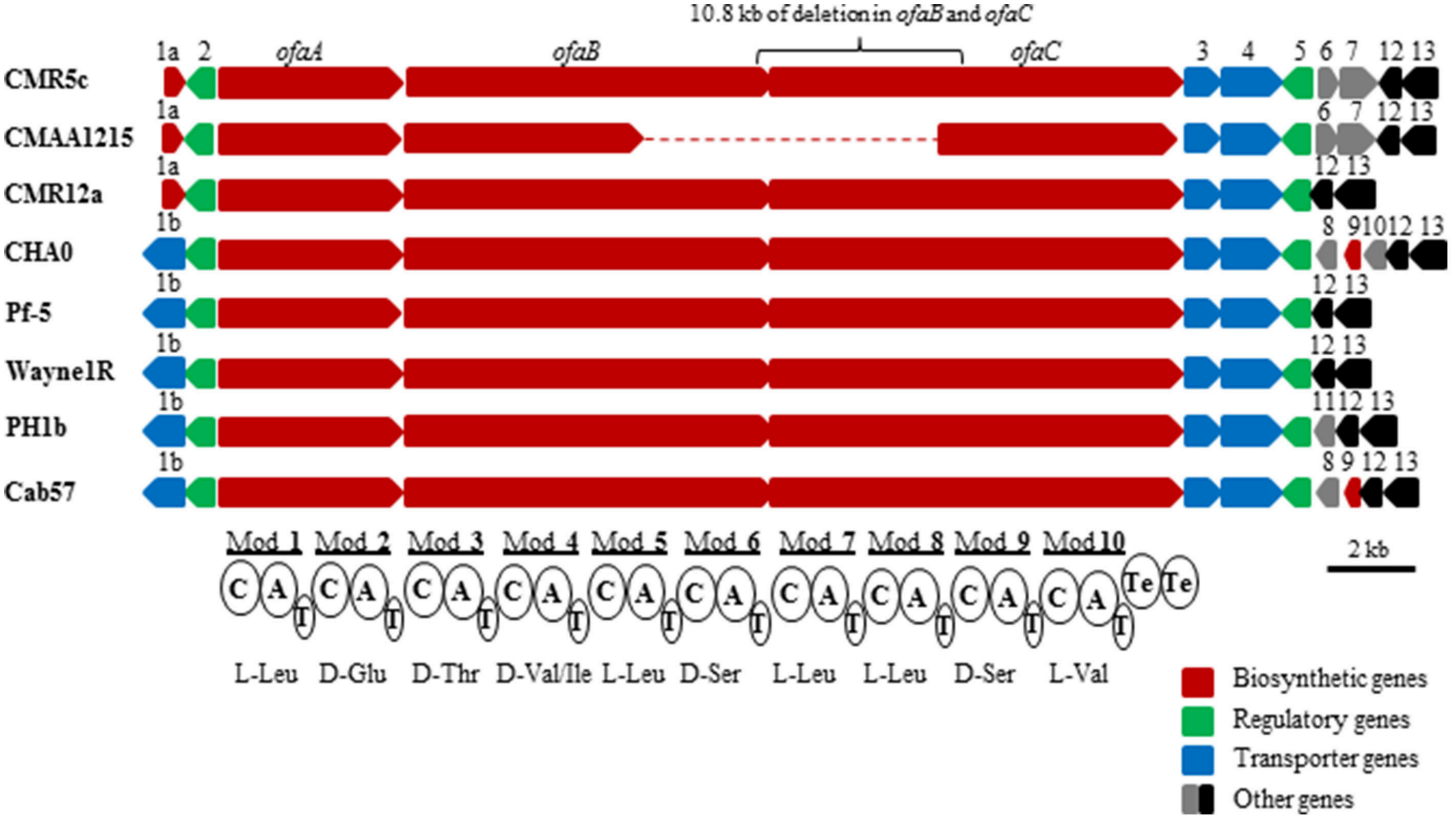
*****In silico*** analysis and comparison of orfamide-synthase gene clusters and flanking regions of ***P. protegens*** and related species**. All orfamide-synthases contain three large structural genes, *ofaA, ofaB*, and *ofaC*; these structural genes synthesize 10 modules for amino acids biosynthesis and two TE domains for peptide release, each module contained three domains, A, condensation (C), and thiolation (T) domains. The detailed interpretation of orfamide-synthases and flanking regions of these strains is shown in Table 2. Only partial gene sequences of orfamide synthases of *Pseudomonas* sp. CMAA1215 could be retrieved from the GenBank database. Scale bar is 2 kb.

Genome mining results showed that three large genes (Figure 1) are present in the whole genome of *Pseudomonas* sp. CMR5c. Results from NRPSpredictor2, NRPS-PKS program, antiSMASH 3.0, and a BLAST search showed these three biosynthetic genes encoded 10 amino acids, similar to those of orfamides from *P*. *protegens* Pf-5 and *Pseudomonas* sp. CMR12a (Gross et al., 2007; D'aes et al., 2014). Moreover, BLAST search revealed that *Pseudomonas fluorescens* Wayne1R, *Pseudomonas* sp. PH1b and *Pseudomonas* sp. CMAA1215 may be potential orfamide-producers. Synthases of reported orfamide-producers (*P*. *protegens* CHA0, Pf-5 and Cab57, *Pseudomonas* sp. CMR12a) were also retrieved from GenBank database and all these orfamide synthases were compared in this study (Gross et al., 2007; Kupferschmied et al., 2013; D'aes et al., 2014; Takeuchi et al., 2014). Orfamide synthases contain three NRPS structural genes designed as *ofaA, ofaB*, and *ofaC*, which respectively encode two, four, and four amino acids (Figure 1). Orfamide-synthases of these *Pseudomonas* strains also contain two terminal thioesterase (TE) domains, which cleave the peptides from the NRPSs (Figure 1). BLASTp comparison of orfamide-synthases genes and flanking regions showed a very high similarity among *P. protegens* isolates, while *Pseudomonas* sp. CMR12a, CMR5c, CMAA1215, and PH1b were more distantly related to *P. protegens*. Intriguingly, orfamide synthases and flanking regions in *Pseudomonas* sp. CMR5c from Cameroon and *Pseudomonas* sp. CMAA1215 from Brazil show a very high similarity (Table 2).

**Table 2:** **Putative orfamide-synthases and flanking region identified from ***P. protegens*** and related strains in this study**.

**Orfamide-synthases and flanking area**		**Identity at amino acid level**							
		***P. protegens***				***Pseudomonas*** **sp**.			
		**CHA0**	**Cab57**	**Pf-5**	**Wayne1R**	**CMR5c**	**CMR12a**	**CMAA1215**	**PH1b**
1a	GCN5-related N-acetyltransferase					[100%]	[75%]	[100%]	
						ALG76232.1	AFH75326.1	ERO60965.1	
1b	RND efflux system, outer membrane lipoprotein	100%	99%	99%	99%				87%
		AGL83955.1	BAO61507.1	AAY91416.1	WP_019094693.1				WP_025130134.1
2	LuxR family transcriptional regulator	100%	99%	100%	99%	80% [100%]	78% [82%]	80% [100%]	88% [80%]
		AGL83956.1	BAO61508.1	AAY91417.1	WP_011060444.1	ALG76233.1	AFH75327.1	ERO60966.1	WP_025130133.1
*ofaA*	Nonribosomal peptide synthase	100%	99%	99%	99%	80% [100%]	79% [84%]	80% ERO60967.1	83% [80%]
		AGL83957.1	BAO61509.1	AAY91419.3	WP_019094694.1	ALG76234.1	AFH75328.1	+78% ERO60968.1	WP_025130132.1
								+87% ERO60969.1	
*ofaB*	Nonribosomal peptide synthase	100%	99%	99%	99%	81% [100%]	82% [89%]	82% [99%]	85% [80%]
		AGL83958.1	BAO61510.1	AAY91420.2	WP_037007783.1	ALG76235.1	AFH75329.1	ERO60970.1^*^	WP_025130131.1
*ofaC*	Nonribosomal peptide synthase	100%	100%	99%	98%	82% [100%]	82% [88%]	80% ERO60971.1	86% [82%]
		AGL83959.1	BAO61511.1	AAY91421.3	WP_019095728.1	ALG76236.1	AFH75330.1	+ERO60972.1^*^	WP_025130130.1
3	Macrolide efflux protein MacA	100%	99%	99%	99%	93% [100%]	92% [97%]	93% [100%]	98% [93%]
		AGL83960.1	BAO61512.1	AAY91422.1	WP_011060449.1	ALG76237.1	AFH75331.1	ERO60973.1	WP_025130129.1
4	Macrolide efflux protein MacB	100%	99%	99%	99%	93% [100%]	91% [97%]	96% [95%]	97% [93%]
		AGL83961.1	BAO61513.1	AAY91423.1	WP_019095727.1	ALG76238.1	AFH75332.1	ERO60957.1^*^	WP_025130128.1
5	LuxR family transcriptional regulator	100%	99%	99%	99%	78% [100%]	74% [81%]	78% [100%]	78% [77%]
		AGL83962.1	BAO61514.1	AAY91424.1	WP_019095116.1	ALG76239.1	AFH75333.1	ERO60958.1	WP_025130127.1
6	Flagellar basal body rod modification protein FlgD					[100%]		99%	
						ALG76240.1		ERO60959.1	
7	Flagellar hook protein FlgE					[100%]		99%	
						ALG76241.1		ERO60960.1	
8	Putative gnat family acetyltransferase	100%	100%						
		AGL83963.1	BAO61515.1						
9	Family 2 glycosyl transferase	100%	100%						
		AGL83964.1	BAO61516.1						
10	Hypothetical protein	AGL83965.1							
11	Hypothetical protein								WP_029978544.1
12	Glyoxalase family protein	100%	100%	100%	99%	82% [100%]	79% [90%]	82% [100%]	91% [80%]
		AGL83966.1	BAO61517.1	AAY91425.1	WP_019095115.1	ALG76242.1	AFH75334.1	ERO60961.1	WP_025130125.1
13	Heme transporter CcmD, radical SAM domain-containing protein	100%	99%	99%	99%	93% [100%]	92% [95%]	93% [99%]	97% [93%]
		AGL83967.1	BAO61518.1	AAY91426.1	WP_026020220.1	ALG76243.1	AFH75335.1	ERO60962.1	WP_025130124.1

*The identity level of amino acids was compared by BLASTp search. The first BLASTp similarity comparison is based on protein sequence of P. protegens CHA0. The second BLASTp similarity comparison is based on Pseudomonas sp. CMR5c and the level of similarity is shown in brackets*.

* *Partial NRPS sequences were retrieved from Pseudomonas sp. CMAA1215 in GenBank database*.

**Figure 2:**
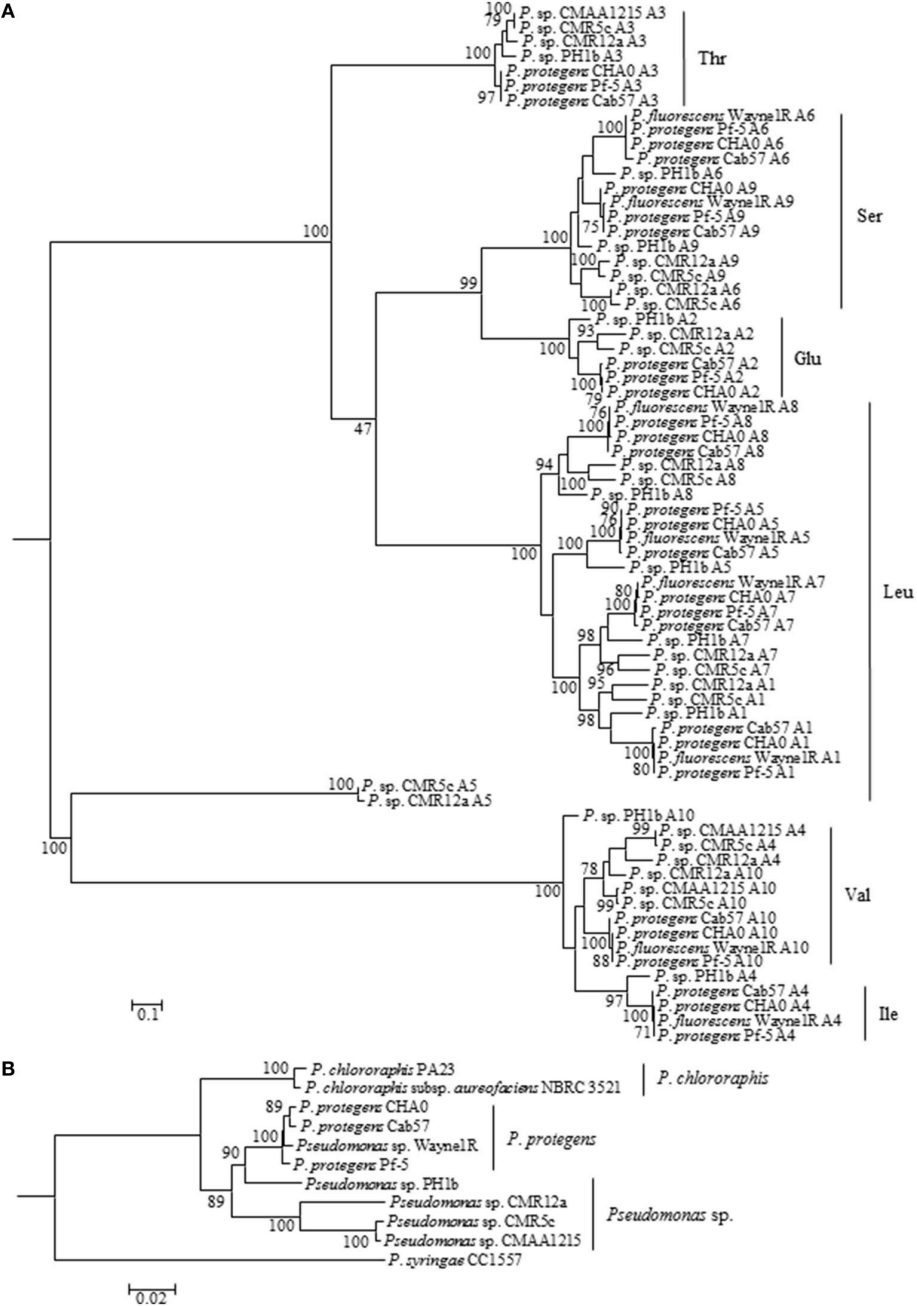
**Molecular phylogenetic (Maximum Likelihood method with 500 of Bootstrap replications) analysis of A domains of orfamide synthases extracted from ***P. protegens*** and related species (A)**. The amino acids were predicted based on comparison of those reported orfamide-synthases in *Pseudomonas* sp. CMR12a and *P*. *protegens* Pf-5. A phylogeny tree was constructed based on *rpoD* and *gyrB* genes of orfamide-producing and related *Pseudomonas* species **(B)**. The tree was constructed with MEGA5 (Maximum Likelihood method with 1000 Bootstrap replications).

In NRPSs, adenylation (A) domains are responsible for recruiting and selecting amino acids for peptide biosynthesis (Strieker et al., 2010). A domains of reported orfamide-producing pseudomonads and the potential orfamide producers *P*. *fluorescens* Wayne1R, and *Pseudomonas* sp. PH1b, CMAA1215, and CMR5c, were retrieved and analyzed by phylogenetic analysis. Domains of orfamide synthases were extracted by NRPSpredictor2. The phylogenetic tree analysis of A domains showed that all these orfamide synthases recruit the same amino acids in each substitution, except for the fourth position. *P*. *protegens, Pseudomonas* sp. PH1b, and *P*. *fluorescens* Wayne1R recruit an isoleucine, while the fourth position in *Pseudomonas* sp. CMR5c, CMR12a, and CMAA1215 encodes a valine (Figure 2A).

Genome mining results showed diversification of the flanking regions of orfamide gene clusters (Figure 1). GCN5-related N-acetyltransferase and *luxR* type transcriptional regulator genes are located upstream of structural synthase genes (*ofaA, ofaB*, and *ofaC*) of *Pseudomonas* sp. CMR5c, CMR12a, and CMAA1215, while a NodT type outer membrane lipoprotein gene and *luxR* type transcriptional regulator genes are located upstream of these structural synthase genes in *P*. *fluorescens* Wayne1R, *P*. *protegens* CHA0, Pf-5, Cab57, and *Pseudomonas* sp. PH1b. Two transporter genes encoding macrolide efflux protein MacA and MacB are located between *ofaC* gene and *luxR* type transcriptional regulator gene in all orfamide gene clusters, although the region downstream of *luxR* type transcriptional regulator gene showed differences. Two genes encoding flagella (flagellar basal body rod modification protein FlgD and flagellar hook protein FlgE) are present in *Pseudomonas* sp. CMR5c and CMAA1215, while two genes encoding a putative GNAT family acetyltransferase and family 2 glycosyl transferase are found in *P*. *protegens* CHA0 and Cab57. A putative glyoxalase family protein and a heme transporter CcmD, radical SAM domain-containing protein are located downstream of the orfamide biosynthetic gene clusters in all strains. The detailed comparison and interpretation is shown in Table 2.

A phylogenetic analysis based on *rpoD* and *gyrB* gene sequences of orfamide-producing *Pseudomonas* species indicated that *P*. *fluorescens* Wayne1R groups with *P*. *protegens*, while *Pseudomonas* sp. PH1b is more distantly related. *Pseudomonas* sp. CMR12a, *Pseudomonas* sp. CMR5c and CMAA1215 belong to a separate group related to *P*. *protegens*. Intriguingly, CMR5c and CMAA1215 showed almost 100% similarity (Figure 2B). The phylogenetic analysis results of A domains from orfamide-synthases appear to coincide well with the house keeping (*rpoD* and *gyrB*) gene analysis and the flanking regions of the orfamide cluster.

### Chemical analysis of orfamides

**Figure 3:**
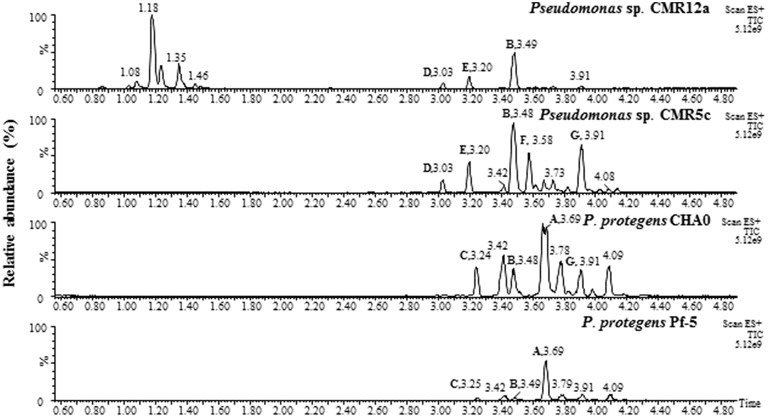
**UPLC-MS analysis of crude extracts from selected ***Pseudomonas*** strains, ***P. protegens*** CHA0 and Pf-5, ***Pseudomonas*** sp. CMR12a and CMR5c**. Samples of Pseudomonas sp. CMR12a were prepared from cells on soft agar plate since orfamide secretion in this strain is hampered by the presence of sessilin (D'aes et al., 2014). Samples of other Pseudomonas strains were prepared from supernatant of overnight liquid KB cultures.

UPLC-MS analysis of samples of *Pseudomonas* sp. CMR5c, CMR12a, and *P*. *protegens* Pf-5 and CHA0 confirmed the presence of orfamides homologs (Figure 3), in agreement with results of bioinformatic characterization of NRPS gene clusters (Figure 1). Retention times from UPLC and MS data of CLPs, together with bioinformatic characterization of CLP synthases revealed that orfamide homologs produced by *P*. *protegens* CHA0 and Pf-5 are exactly the same (Figures 2A, 3, Table 3 and Supplementary Table 2). Furthermore, the retention time and mass data of peaks from *Pseudomonas* sp. CMR5c were compared with those of reported orfamide homologs, such as orfamide A-E (Gross et al., 2007; D'aes et al., 2014). Interestingly, two new peaks were found in the KB supernatant of *Pseudomonas* sp. CMR5c, and those peaks were designed as orfamide F (*m*/*z* [M+H]^+^ of 1307.7) and orfamide G (*m*/*z* [M+H]^+^ of 1309.6). These masses were further confirmed by LC-MS analysis (Supplementary Table 2). The difference of two mass units between orfamide F and orfamide G suggests an additional unsaturation, such as a double bond, in orfamide F.

**Table 3:** **Structure of orfamide derivatives isolated and characterized from ***P. protegens*** and related strains**.

**Compound**	**Fatty acid residue**	**Amino acid sequence**										**Producing strains**
		**1**	**2**	**3**	**4**	**5**	**6**	**7**	**8**	**9**	**10**	
Orfamide A	C14:0-OH (3)	L-Leu	D-Glu	D-aThr	D-Ile	L-Leu	D-Ser	L-Leu	L-Leu	D-Ser	L-Val	CHA0, Pf-5
Orfamide B	C14:0-OH (3)	L-Leu	D-Glu	D-aThr	D-Val	L-Leu	D-Ser	L-Leu	L-Leu	D-Ser	L-Val	CMR12a, CMR5c, CHA0, Pf-5
Orfamide C	C12:0-OH (3)	L-Leu	D-Glu	D-aThr	D-Ile	L-Leu	D-Ser	L-Leu	L-Leu	D-Ser	L-Val	CHA0, Pf-5
Orfamide D	C12:0-OH (3)	L-Leu	D-Glu	D-aThr	D-Val	L-Leu	D-Ser	L-Leu	L-Leu	D-Ser	L-Val	CMR12a, CMR5c
Orfamide E	C14:1-OH (3)	L-Leu	D-Glu	D-aThr	D-Val	L-Leu	D-Ser	L-Leu	L-Leu	D-Ser	L-Val	CMR12a, CMR5c
Orfamide F	C16:1-OH (3)	L-Leu	D-Glu	D-aThr	D-Val	L-Leu	D-Ser	L-Leu	L-Leu	D-Ser	L-Val	CMR5c
Orfamide G	C16:0-OH (3)	L-Leu	D-Glu	D-aThr	D-Val	L-Leu	D-Ser	L-Leu	L-Leu	D-Ser	L-Val	CMR12a, CMR5c, CHA0, Pf-5

*The new orfamides F and G were isolated and characterized from Pseudomonas sp. CMR5c. All orfamides from Pseudomonas strains share an unbranched β-hydroxyl fatty acid chain that is saturated for all homologs, except for orfamides E and F, which contain a single double bond within the fatty acid chain. Orfamides A and C possess a valine in the fourth position, as opposed to the other orfamides possessing an isoleucine at the fourth position*.

The orfamides produced by *Pseudomonas* sp. CMR5c were subjected to further chemical identification. Crude extracts from precipitates of KB supernatant of *Pseudomonas* sp. CMR5c were separated on a SPE C18 cartridge. The eluents of 80% (v/v) and 100% (v/v) acetonitrile were active in a droplet collapse assay. In these fractions, the presence of orfamides was confirmed by UPLC-MS and they were collected and dried. Semi-purified orfamides were further separated on a RP-HPLC system and finally yielded three purified compounds, **1** (14.0 min, 33.54 mg), **2** (14.8 min, 9.4 mg), and **3** (20.9 min, 5.8 mg), respectively.

### Chemical structure elucidation of new orfamides

The ^1^H-NMR spectra of compounds **1**, **2**, and **3** showed signal patterns characteristic of a peptide (Supplementary Table 3). Ten amino acids, including Leu (4 ×), Glu (1 ×), Thr (1 ×), Ser (2 ×), Val (2 ×), were identified by ^1^H-^1^H correlation spectra of COSY and TOCSY, while their position in the sequence was determined through a combination of the ROESY and ^1^H-^13^C HMBC spectra. All three compounds contained the same peptide backbone: Leu^1^Glu^2^Thr^3^Val^4^Leu^5^Ser^6^Leu^7^Leu^8^Ser^9^Val^10^. The cyclization of **1** is directly demonstrated by the presence of a ^3^J_CH_ HMBC correlation between the C-terminal Val_10_ carbonyl ^13^C and the Thr_3_ H^β^ resonances. The ^1^H-NMR spectrum of compound **2** showed a resonance at 5.35 ppm that integrated for 2 protons and correlated with a CH-type carbon at 130.13 ppm, indicating the presence of an alkene function. Analysis of the COSY, TOCSY, and ^1^H-^13^C HMBC spectra of **2** showed correlations from and to these ^1^H and ^13^C resonances with a large pool of protons at ca. 1.3 ppm, characteristic of the fatty acid chain and confirming the presence of the double bond in this moiety, however, the exact position of double bond in the fatty acid residue of compound **2** could not be determined. The ^13^C chemical shifts of the neighboring CH_2_'s suggest a *cis* configuration for this double bond (Supplementary Table 3) (Li et al., 2013). Compounds **1** and **3** contained normal (fully saturated) fatty acid moieties. Since the full peptide sequence has been established, the length of these linear moieties can be deduced from the mass obtained from MS, confirming a C_14_ chain length for compound **1** and C_16_ chain length for **2** and **3**. Compound **1** was thus identified as orfamide B, while **2** and **3** were identified as new CLPs, dubbed orfamide F and orfamide G respectively (Table 3). The NMR assignment of these compounds is given in Supplementary Table 3. UPLC-MS data revealed that the first two main compounds produced by *Pseudomonas* sp. CMR5c have the same molecular weight and retention time as orfamide D and orfamide E from *Pseudomonas* sp. CMR12a (Figure 3). Moreover, considering that the domain analysis result of orfamide biosynthetic synthase showed high similarities between these two strains, it is reasonable to conclude that the first two main compounds produced by *Pseudomonas* sp. CMR5c may be assigned as orfamide D and E, respectively (Table 3).

### Orfamides determine the surface swarming motility of *Pseudomonas* sp. CMR5c and their production is regulated by luxR-type regulators

**Figure 4:**
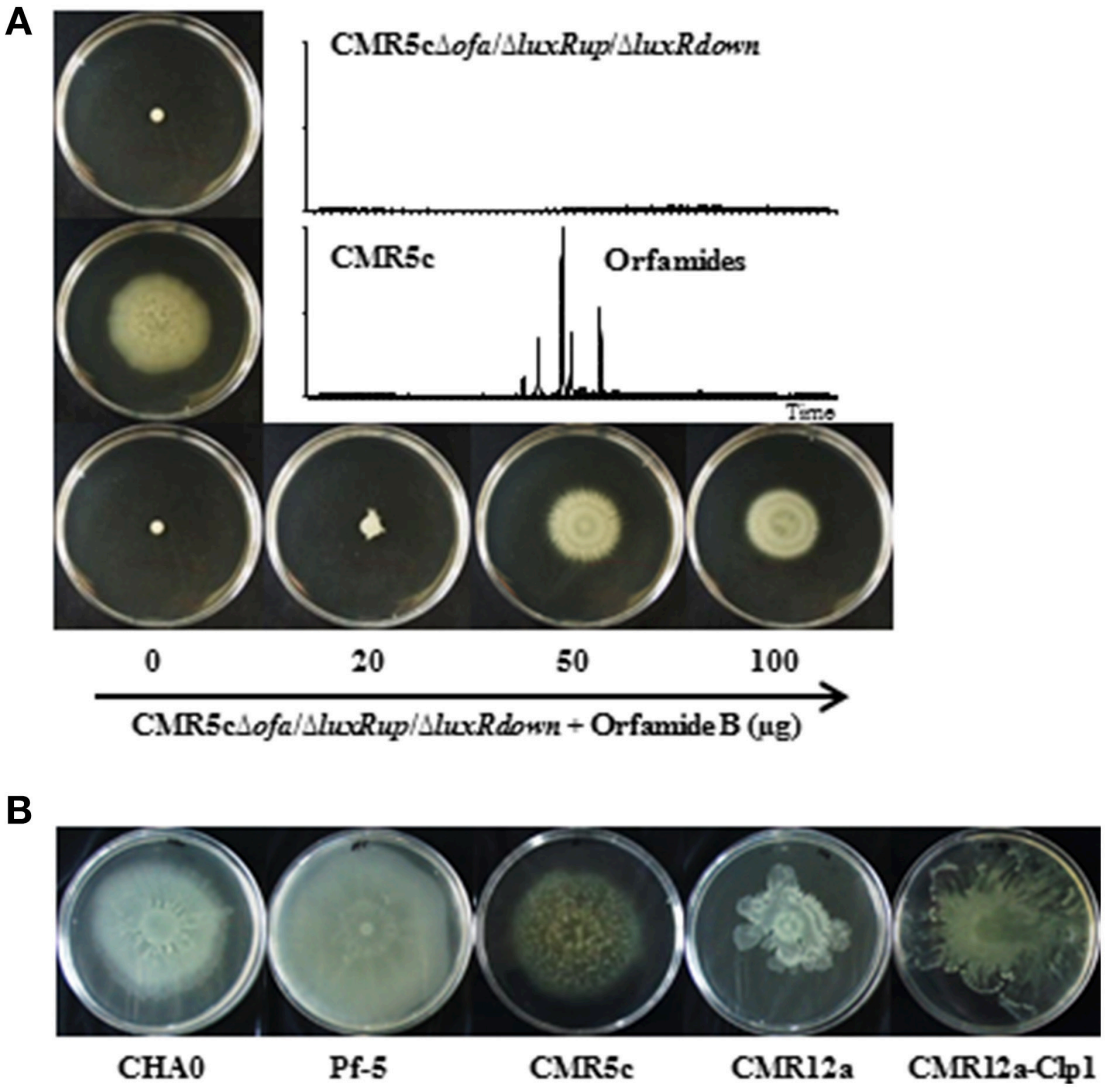
**(A)** Swarming motility of wild type strain *Pseudomonas* sp. CMR5c and deletion mutants in orfamide biosynthesis (CMR5cΔ*ofa*) or in orfamide regulatory genes (CMR5cΔ*luxRup* and CMR5cΔ*LuxRdown*). In all mutants swarming was restored by chemical complementation with orfamide B. Representative plates are shown and the diameter of soft agar plates used in this assay is 9 cm. The insert shows the UPLC-MS analysis of wild type and mutant strains. **(B)** Swarming motility of *P. protegens* CHA0, *P. protegens* Pf-5, *Pseudomonas* sp. CMR5c*, Pseudomonas* sp. CMR12a, and its sessilin mutant CMR12a-Clp1.

To study the function of *ofa* genes and *luxR* genes of *Pseudomonas* sp. CMR5c, a 10.8 kb fragment was deleted from *ofaB* and *ofaC* genes (Figure 1), a 661 bp fragment was deleted from luxR-type transcriptional regulator gene (*luxRup*) located upstream of structural genes of orfamide synthase, and a 441 bp fragment was deleted from *luxR* type transcription regulator gene (*luxRdown*) located downstream of structural genes of orfamide synthase, generating *Pseudomonas* sp. CMR5cΔ*ofa*, CMR5cΔ*luxRup*, and CMR5cΔ*luxRdown* mutants (Table 1). UPLC-MS data showed that the production of orfamide homologs was abolished in the orfamide biosynthesis mutant *Pseudomonas* sp. CMR5cΔ*ofa* (Figure 4A). Similar results were found for the two *luxR*-type transcriptional regulator mutants CMR5cΔ*luxRup* and CMR5cΔ*luxRdown*. Furthermore, phenotypic characterization showed that in all three mutants swarming motility was blocked compared to wild type *Pseudomonas* sp. CMR5c strain (Figure 4A). Chemical complementation experiments were conducted with soft agar plates amended with different concentrations of orfamide B. The results showed that in all three mutants the swarming phenotype was restored by adding orfamide B in soft agar plates (Figure 4A). The swarming motility of orfamide-deficient mutants of *Pseudomonas* sp. CMR5c showed a dose-dependent response, that is, bacterial motility increased on soft agar plates containing higher concentrations of orfamides.

Model strains *P*. protegens CHA0 and Pf-5, *Pseudomonas* sp. CMR5c, and CMR12a and its sessilin mutant *Pseudomonas* sp. CMR12a-Clp1 were included in further phenotypic comparison. The sessilin mutant was included because previous work has shown that the presence of sessilin hampers orfamide secretion in *Pseudomonas* sp. CMR12a (D'aes et al., 2014). All these strains could swarm well on the agar surface, suggesting at least a partial involvement of orfamides in bacterial motility (Figure 4B).

### *In vitro* antibiosis activity against *R. solani* and *oomycete* pathogens

**Figure 5:**
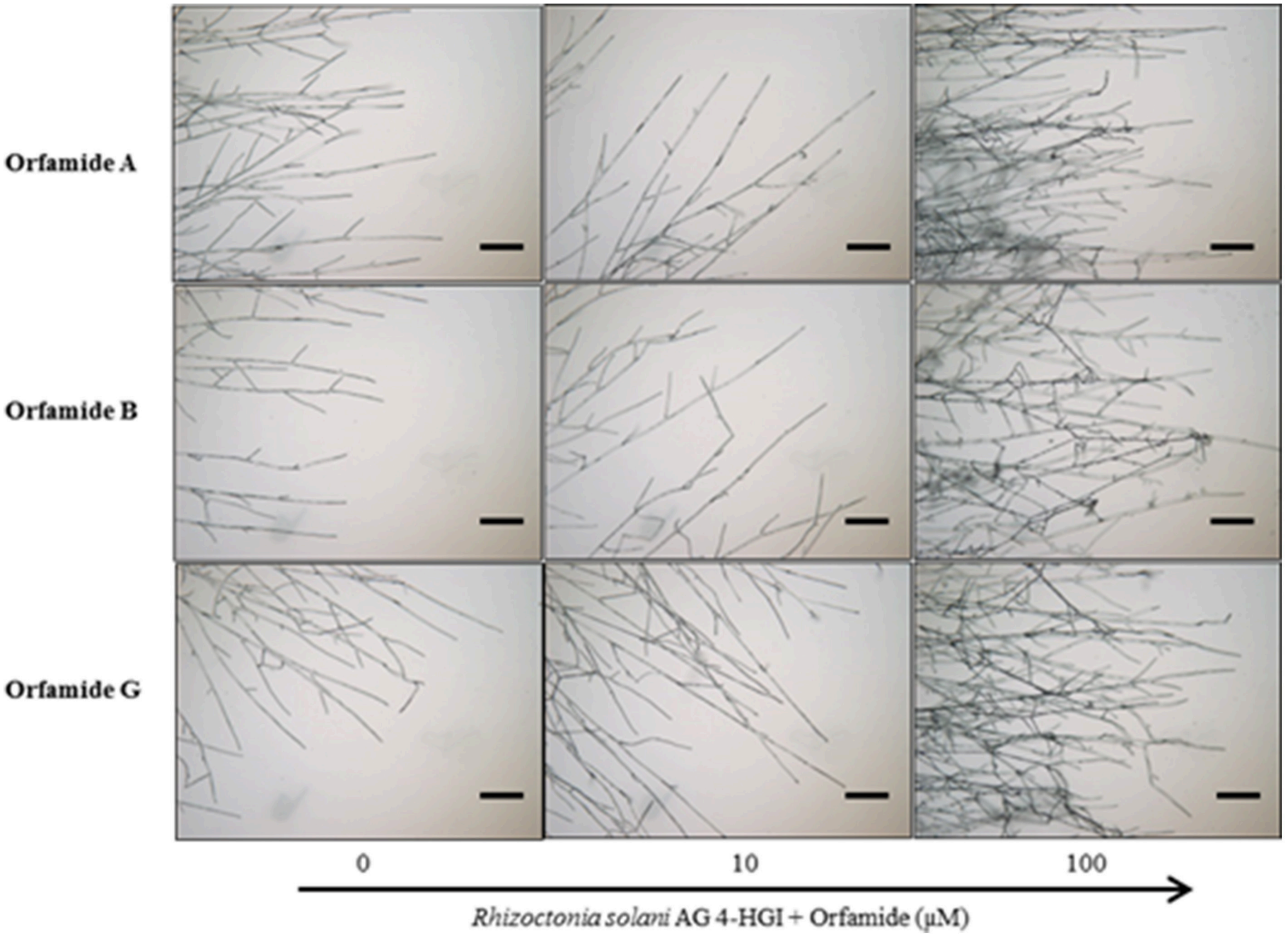
**Microscopic assays showing the effect of various concentrations of orfamides (Orfamide A, orfamide B, and orfamide G) on hyphal branching of ***Rhizoctonia solani*** AG 4-HGI, scale bar = 100 μm**. Sterile microscopic glass slides were covered with a thin, flat layer of water agar (Bacto agar; Difco) and placed in a plastic Petri dish containing moist sterile filter paper. An agar plug (Diameter = 5 mm) taken from an actively growing colony of *R. solani* was inoculated at the center of each glass slide. Two droplets (15 μl each) containing 0, 10, or 100 μM orfamide were placed at two sides of the glass slide at about 2 cm from the fungal plug. Slides were incubated for 36 h at 28°C before evaluation under an Olympus BX51 microscope.

**Figure 6:**
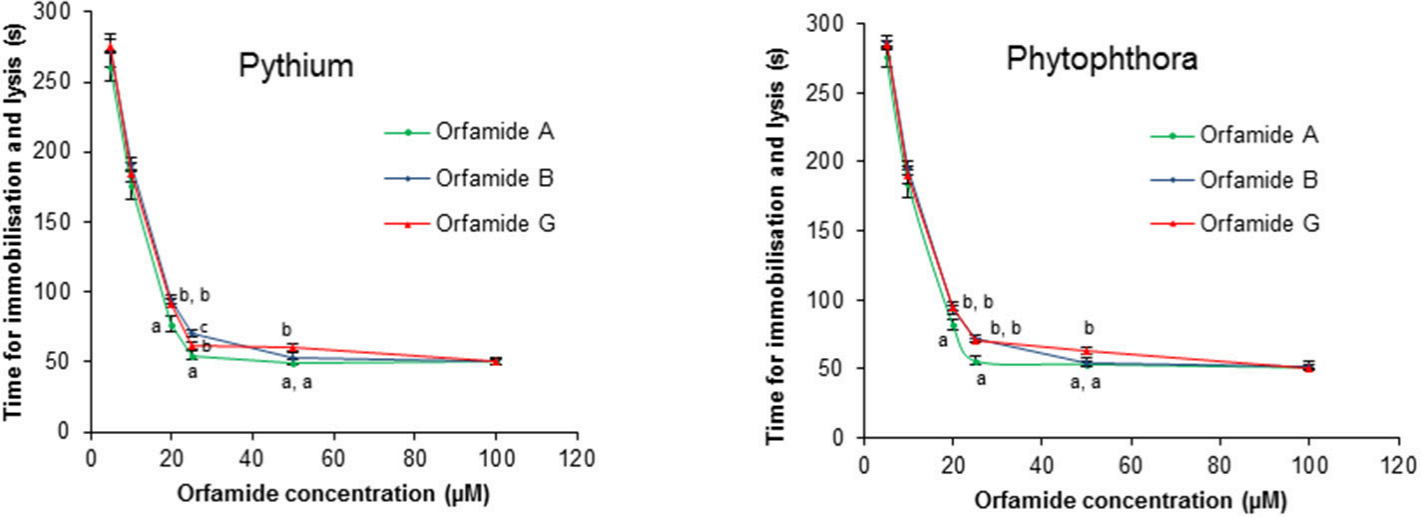
**Effect of orfamides (orfamide A, orfamide B, and orfamide G) on the viability of zoospores of the oomycete pathogens ***Pythium ultimum*** and ***Phytophthora porri*** CBS 127099 (B)**. Zoospores of both pathogens were incubated in the presence of increasing concentrations of orfamide A and B and the time (in seconds) that it took to observe lysis was recorded immediately under an Olympus BX51 microscope. Data are the mean of three repetitions. Vertical bars indicate standard deviations. Different letters indicate significant differences among different treatments for that specific concentration (Tukey's test, α = 0.05).

Orfamide A produced by *P*. *protegens* CHA0, and orfamide B and G produced by *Pseudomonas* sp. CMR5c, were compared in further bioassays. Orfamide A and orfamide B differ only by an amino acid substitution at the fourth position: a valine in orfamide A and an isoleucine in orfamide B; orfamide B and orfamide G share the same amino acid sequence but differ in length of the fatty acid chain, a C_14_ for orfamide B and C_16_ for orfamide G (Table 3). Previous results showed that orfamide B suppressed mycelial growth and caused increased hyphal branching of *R. solani* AG 4-HGI at 100 μM (Olorunleke et al., 2015b). Moreover, orfamide A showed zoospores lysis activity against *Phytophthora ramorum* Pr-102 (Gross et al., 2007). We wished to assess whether the subtle structural difference between orfamide A, B, and G would influence these activities. The three orfamides caused increased hyphal branching of *R. solani* AG 4-HGI at 100 μM, while lower concentrations were not effective (Figure 5). At concentrations of 25 μM or higher all orfamides could lyse zoospores of *P. porri* CBS 127099 and *P. ultimum* within 55–70 s. At concentrations of 20 and 25 μM, orfamide A was slightly faster in causing zoospore lysis than the two other orfamides (Figure 6).

### Orfamides inhibit appressoria formation in *M. oryzae*

**Figure 7:**
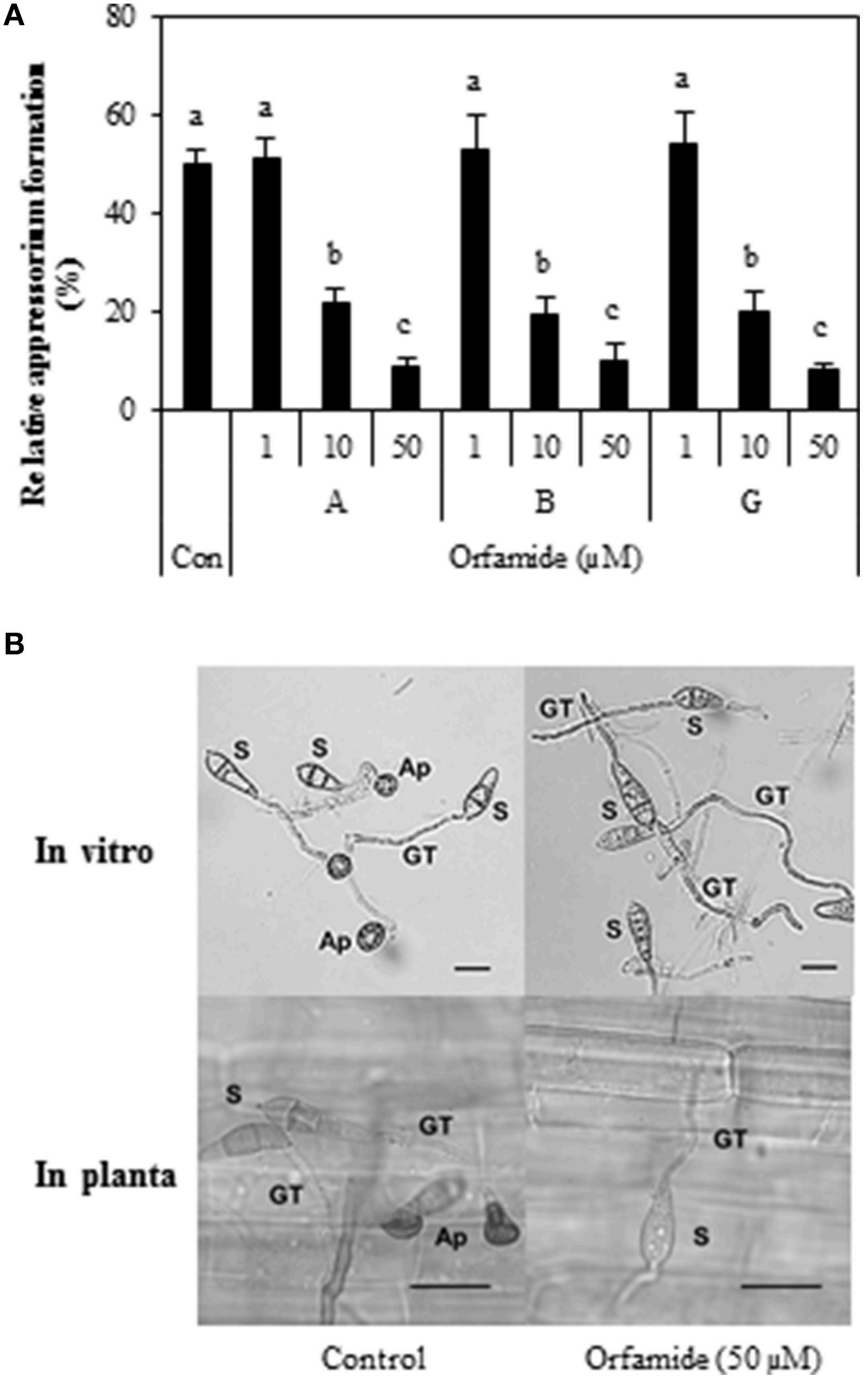
**(A)** Effect of different concentrations (1, 10, and 50 μM) of orfamides (orfamide A, B, and G) on appressorium formation in *Magnaporthe oryzae* VT5M1. The control treatment (Con) received the same amount of DMSO as the orfamide treatments. Data are shown as mean value (±*SD*) for three biological repeats. Different letters indicate significant differences among different treatments (Tukey's test; α = 0.05). **(B)** Representative pictures of appressoria formation in *M*. *oryzae* in control (DMSO) and orfamide treatments after 8 h incubation (*in vitro* assay) or 24 h incubation (in planta assay). S, spore; GT, germ tube; Ap, appressorium. Scale bar is 20 μm.

Subsequently, we tested whether orfamides would have an effect against the rice blast pathogen *M. oryzae*. An *in vitro* spore germination assay with *M. oryzae* isolate VT5M1 showed that both orfamide-treated and control treated spores had the same level of germination, with 92.1% ± 3.1 for the control, 90.1% ± 2.6 for orfamide A (50 μM), 91.7% ± 4.6 for orfamide B (50 μM), and 93.1% ± 3.2 for orfamide G (50 μM). Orfamides (Orfamide A, B, and G) did not show any antagonistic effect against *M. oryzae* isolate VT5M1 in an *in vitro* paper-agar disc diffusion assay (data not shown). However, the three compounds actively blocked appressoria formation in *M. oryzae* in a dose dependent manner (Figure 7A). Representative microscopic pictures showing the effect of orfamide A on appressorium formation are shown in Figure 7B. Appressorium formation *in vivo* was not quantified, but no appressoria were formed in the treatment with 50 μM orfamide in two independent repeats.

### Orfamides reduce blast disease severity on rice plants

**Figure 8:**
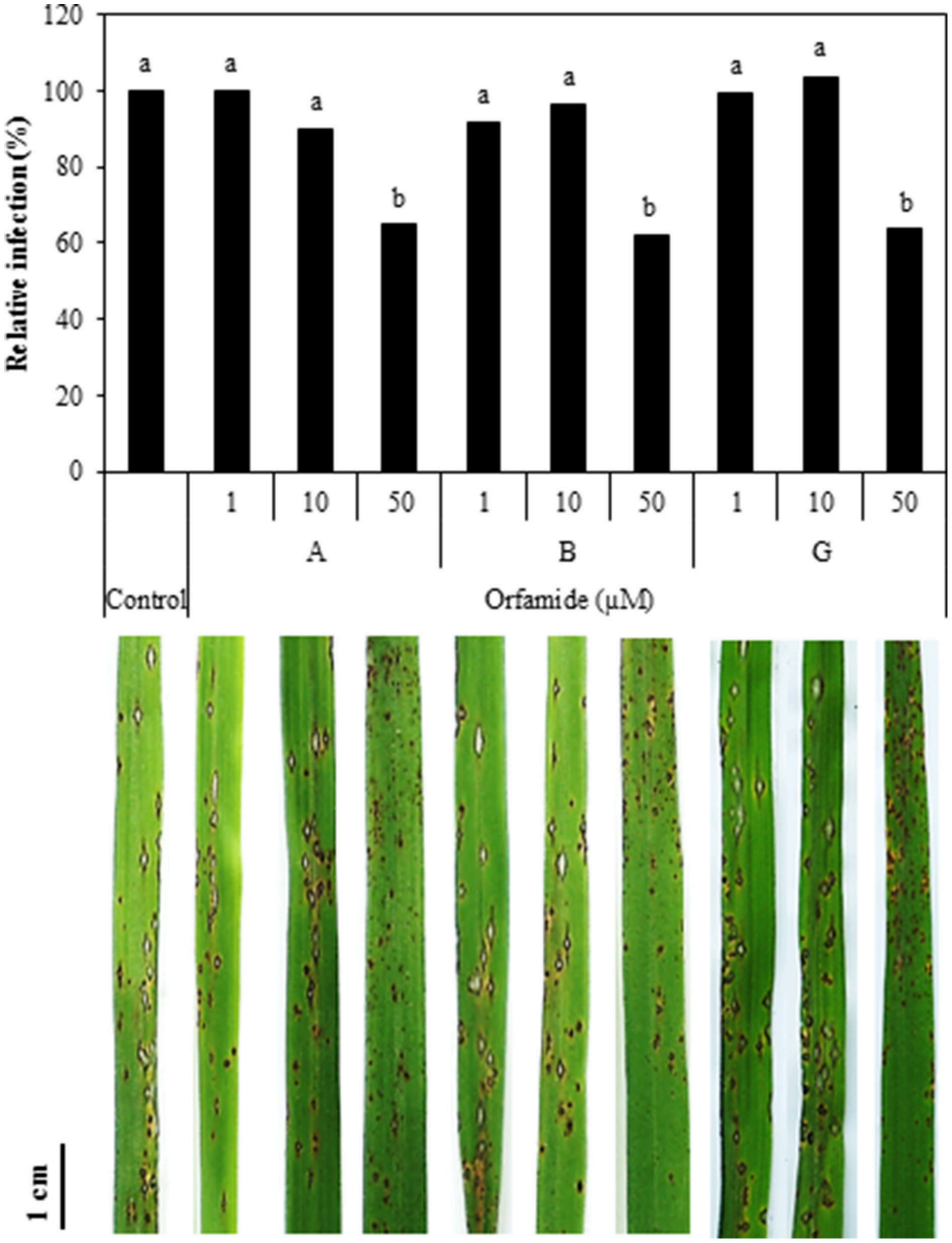
**Influence of orfamides (Orfamide A, B, and G) on rice blast symptoms caused by ***M. oryzae*** isolate VT5M1**. Spores of *M. oryzae* were mixed with different concentrations of orfamides and sprayed on rice plants at the five-leaf stage. Disease was assessed 6 days post infection by counting the number of sporulating susceptible-type lesions on the fourth leaf of rice plants and expressed relative to control plants. Biocontrol assays were repeated independently for three times and showed similar results, data from one representative experiment are shown. Different letters indicate significant differences among different treatments (Mann-Whitney: *n* = 24; α = 0.05). Pictures show representative disease symptoms in the different treatments. Scale bar is 1 cm.

Different concentrations of orfamide A, B, and G were mixed with spores of *M. oryzae* isolate VT5M1 and sprayed on rice plants (Five-leaf stage) and disease symptoms were evaluated after 6 days. All compounds reduced the number of sporulating susceptible-type blast lesions at a concentration of 50 μM, while lower concentrations were not effective (Figure 8).

## Discussion

This study shows that *Pseudomonas* sp. CMR5c produces orfamide-type CLPs, including two new orfamide homologs that we termed orfamide F and orfamide G. Orfamide production appears to be associated with *P*. *protegens* and taxonomically related species. Orfamide producers are plant associated *Pseudomonas* species isolated from the rhizosphere of both dicots and monocots including tobacco (*P*. *protegens* CHA0) (Stutz et al., 1986), cotton (*P. protegens* Pf-5) (Howell and Stipanovic, 1979), shepherd's purse (*P. protegens* Cab57) (Takeuchi et al., 2014), cocoyam (*Pseudomonas* sp. CMR12a and CMR5c) (Perneel et al., 2007), and corn (*P*. *fluorescens* Wayne1R) (Rong et al., 2012). Moreover, also *Pseudomonas* sp. PH1b derived from the phytotelma (=water body) of a carnivorous plant and *Pseudomonas* sp. CMAA1215 (Vasconcellos et al., 2013) from Brazilian mangroves have the genetic information to produce orfamides. Phylogenetic analysis based on the housekeeping genes *rpoD* and *gyrB* shows that *P. fluorescens* Wayne1R (a rifampicin resistant variant of *P. fluorescens* Wayne1) groups with *P*. *protegens* (Figure 2B) while *Pseudomonas* sp. PH1b is more distantly related to *P. protegens*. It was already shown by Redondo-Nieto et al. (2013) based on a phylogenomic analysis that *P. fluorescens* Wayne1 is closely related to *P. protegens* Pf-5. *Pseudomonas* sp. CMR12a, CMR5c, and CMAA1215 clearly belong to a distinct phylogenetic group. Strikingly, however, is the very close relationship between *Pseudomonas* sp. CMR5c from Cameroon and *Pseudomonas* sp. CMAA1215 from Brazil. These two isolates probably have their ecological niche in common, namely a tropical area with periods of high humidity alternating with dry periods.

Bioinformatic analysis of the orfamide gene clusters shows that orfamide biosynthesis genes and flanking regions closely follow the taxonomic relationship among the isolates. This suggests that the orfamide gene cluster is ancestral and not obtained by horizontal gene transfer. Orfamide biosynthesis genes and flanking regions in *P. protegens* are highly conserved and isolates belonging to this species secrete orfamide A as the main CLP, which was also confirmed by chemical analysis for *P. protegens* CHA0 and Pf-5. Bioinformatic analysis results showed that the more distantly related isolate *Pseudomonas* sp. PH1b probably also produces orfamide A. *Pseudomonas* sp. CMR12a and CMR5c produce orfamide B as their main CLP. Since *Pseudomonas* sp. CMR5c and CMAA1215 are very similar in their nonribosomal peptide synthases and flanking region (Figure 2A and Table 2) is very likely that *Pseudomonas* sp. CMAA1215 also produces orfamide B.

Most *Pseudomonas* NRPS obey the “colinearity rule,” which means the NRPS structural genes are linked together without disruption by other genes, although viscosin group (WLIP and massetolides, etc.), entolysin group and xantholysin group NRPS peptides do not (de Bruijn et al., 2007, 2008; Vallet-Gely et al., 2010; Li et al., 2013). The orfamide-synthases compared in this study do seem to follow this rule (Figure 1). The structural identification showed that co-produced orfamides not only differ from each other in amino acid sequence, but also in fatty acid residue, with variable length and degree of unsaturation (Table 3). A single orfamide-producer can synthesize orfamides containing both saturated and unsaturated fatty acid chains (*Pseudomonas* sp. CMR12a and CMR5c). *P*. *protegens* can incorporate different amino acids by the same A domain (Figures 2A, 3 and Table 3). For instance, the fourth A domain can incorporate both valine and isoleucine, although in this species the incorporation of isoleucine seems to be preferred, since orfamide A is most abundantly produced. The fourth A domain of *Pseudomonas* sp. CMR12a seems to be less flexible since these strains only produce orfamides with a valine at the fourth position. Flexibility in the A domain has also been observed for putisolvin biosynthesis. The 11th A domain of putisolvin-synthase can use valine/isoleucine/leucine, but the production of putisolvin I containing valine is preferred (Dubern et al., 2008). The mechanisms of this type of synthesizing and catalyzing remain unclear. However, the fact that a single domain can activate two or more different amino acids, leads to difficulties in deducing amino acid sequence only by bioinformatic analysis of peptide synthase in *Pseudomonas* species.

The amount of semi-purified orfamides that can be obtained from *P*. *protegens* CHA0 is much higher than for Pf-5, *Pseudomonas* sp. CMR5c and CMR12a in liquid KB medium (data not shown). This could point to differences in secretion or regulation of these compounds in the various strains. A NodT type outer membrane lipoprotein is located upstream of orfamide-synthases of *P*. *protegens* and *Pseudomonas* sp. PH1b, but not in *Pseudomonas* sp. CMR12a, CMR5c, and CMAA1215. Together with the MacA and MacB encoding genes downstream of the orfamide synthases, these lipoproteins are probably involved in orfamide transport across the inner and outer membrane, but this needs to be confirmed experimentally. Moreover, flanking regions of *Pseudomona*s NRPS structural genes contain one or more *luxR* or *luxR*-like transcriptional regulator genes that control lipopeptide biosynthesis (de Bruijn and Raaijmakers, 2009). Accordingly, this study also shows that orfamide production was blocked in *Pseudomonas* sp. CMR5c when *luxR* type transcriptional regulator encoding genes located at the flanking region of orfamide-synthase were disrupted (Figure 4A). It should be noted however that lipopeptide regulation in *Pseudomonas* is very complex. Recently it was shown that chaperone protein ClpA together with the serine protease ClpP regulated massetolide biosynthesis in *P. fluorescens* SS101 via the LuxR-type transcriptional regulator MassAR, the heat shock proteins DnaK and DnaJ and via proteins involved in the TCA cycle (Song et al., 2015). It is not unlikely that strain-dependent differences in this regulatory circuit account for the differences in orfamide production that we observed in the tested strains.

The structural differences among orfamide A, orfamide B, and orfamide G allowed us to study whether the difference in amino acid at the fourth position and the length of fatty acid residue has any effect on the biological activity. In case of iturin-type *Bacillus* lipopeptides it has been shown that biological activity against fungal pathogens increases with the number of carbon atoms in β-amino fatty acid chain (Tanaka et al., 2014). The defense-inducing activity of surfactin-type *Bacillus* lipopeptides was reduced for some amino acid substitutions and completely lost for surfactins with fatty acid chains shorter than 14 carbons (Henry et al., 2011). In our work, however, the residue in position 4 or the length of the fatty acid chain had no obvious influence on biological activity against Oomycete or fungal pathogens. Microscopic assays showed that all orfamides increased hyphal branching of *R. solani* AG 4-HGI (Figure 5), indicative of mycelial growth inhibition (Olorunleke et al., 2015b). These orfamides however did not show any antibiotic activity against *R. solani* AG 4-HGI by the paper disc-agar diffusion assay as described by Gross et al. (2007). These data confirm the positive results obtained with orfamide B in *Rhizoctonia* branching (Olorunleke et al., 2015b) and the negative results obtained with orfamide A in paper-agar disc diffusion assay published previously (Gross et al., 2007) and indicate that different methods for testing *in vitro* antibiosis activity may lead to different results. It has been shown before that the CLP viscosinamide co-incubated with *R. solani* changed the morphology of the hyphae of this fungal pathogen; hyphae were highly branched compared with control treatments (Thrane et al., 1999; Raaijmakers et al., 2010). At concentrations of 25 μM or higher, orfamides (Orfamide A, B, and G) caused zoospore lysis of *P. porri* CBS 127099 and *P. ultimum* within 55–70 s (Figure 6). Orfamide A was slightly faster in lysing zoospores at concentrations of 20 and 25 μM than the two other orfamides, but the difference is subtle and was not observed at lower or higher concentrations. It has been reported before that orfamide A and the viscosin family CLPs WLIP and viscosinamide, are able to lyse zoospores of the oomycete pathogen *P. ramorum* (Thrane et al., 2000; de Bruijn et al., 2007; Gross et al., 2007).

Blast disease, caused by the filamentous ascomycete fungus *M. oryzae*, is one of the major diseases in rice (Dean et al., 2012) and the productivity of rice is threatened by this pathogen worldwide. *M. oryzae* can directly penetrate the plant cuticle and cell wall of rice by means of appressoria in which a turgor pressure is build up. Inhibition of appressoria formation in *M. oryzae* can significantly reduce its pathogenicity in plants (Liu et al., 2011). The orfamides tested (orfamide A, B, and G) were equally active in inhibiting appressoria formation at 10 and 50 μM and reduced the number of susceptible blast lesions on rice at 50 μM. Spence et al. (2014) already revealed that *P*. *protegens* CHA0 can inhibit appressorium formation of *M. oryzae*, but in their study the corresponding metabolite and underlying mechanism were not elucidated. Intriguingly, biosurfactants such as mannosylerythritol lipids and the synthetic surfactant Tween 20 also blocked appressoria formation by *M. oryzae* on a hydrophobic substrate, but failed to protect rice against blast disease attack. Symptoms on rice leaves caused by *M. oryzae* were neither suppressed nor enhanced by the mannosylerythritol lipids treatment while Tween 20 treatment even increased rice blast severity (Yoshida et al., 2015). These authors hypothesized that inhibition of appressorium formation may be due to changes in surface hydrophobicity by treatment with the surfactant and that appressorium formation is probably not inhibited on the leaf surfaces. In the case of orfamides, however, it appears that appressorium formation is also inhibited on rice leaves. It remains to be investigated whether this is due to effects on surface hydrophobicity or to direct effects of orfamides on appressorium development. Induced resistance can be excluded as a mechanism explaining the effects of orfamide on rice blast. Rice roots inoculated with *Pseudomonas* sp. CMR5c were as susceptible to *M. oryzae* as control plants and soil drench with purified orfamides did not induce resistance to the fungus (Ma and Hofte, unpublished results). Likewise, root inoculation with *P*. *protegens* CHA0 did not result in induced resistance against *M. oryzae* in rice plants (Spence et al., 2014).

Although, orfamides do not induce resistance against *M. oryzae*, we recently found that orfamides can trigger defense-related responses in rice cell cultures and induce resistance against the necrotrophic fungus *Cochliobolus miyabeanus* in rice plants (Ma and Hofte, unpublished results). We are currently investigating this interaction in more detail.

## Author contributions

ZM and MH designed the research; MH supervised the study; MO conducted UPLC-MS analysis; ZM, MO, and NG conducted RP-HPLC separation; NG conducted NMR analysis; ZM performed the rest of the experiments, analyzed data and wrote the paper together with MH. All authors revised the manuscript and approved the final version for submission.

## Funding

ZM is funded by scholarships from China Scholarship Council (CSC, No.201204910376) and a special research fund (Bijzonder Onderzoeksfonds, BOF) from Ghent University. The Research Foundation–Flanders (FWO–Vlaanderen) is acknowledged for a postdoctoral fellowship and a research grants to DS (1.5.133.13N) and JM (G.0901.10 and G.0422.13). JM acknowledges Ghent University for a 4-year BOF research grant to NG. The 500 MHz NMR equipment was funded by the Hercules Foundation (AUGE09/006).

### Conflict of interest statement

The authors declare that the research was conducted in the absence of any commercial or financial relationships that could be construed as a potential conflict of interest.
