# Supplementary material for: Biosynthesis, Chemical Structure, and Structure-Activity Relationship of Orfamide Lipopeptides Produced by Pseudomonas protegens and Related Species
Source: Front Microbiol. 2016 Mar 30;7:382. doi: 10.3389/fmicb.2016.00382 (PMC4811929; doi:10.3389/fmicb.2016.00382)
Supplement: Supplementary file 1 [file DataSheet1.DOCX]

# Supplementary information

**Biosynthesis,** **chemical** **structure** **and** **structure-activity** **relationship** **of** **orfamide** **lipopeptides** **produced** **by** ***Pseudomonas*** ***protegens*** **and** **related** **species**

Zongwang Ma,^1^ Niels Geudens,^2^ Nam Phuong Kieu,^1^ Davy Sinnaeve,^2^ Marc Ongena,^3^ José C. Martins,^2^ Monica Höfte^1^*

^1^ Laboratory of Phytopathology, Faculty of Bioscience Engineering, Ghent University, Ghent, Belgium

^2^ NMR and Structure Analysis Unit, Department of Organic and Macromolecular Chemistry, Ghent University, Ghent, Belgium

^3^ Microbial Processes and Interactions Unit, Faculty of Gembloux Agro-Bio Tech, University of Liège, Gembloux, Belgium

Author for correspondence (Monica Höfte)

monica.hofte@ugent.be

Tel: +32 9 264 60 17

Fax: +32 9 264 62 38

**Supplementary Table** **1** Primer pairs used in this study

| **Name** | **Gene** | **Fragment** | **Primer (5' to 3')^a^** |
| --- | --- | --- | --- |
| CMR5c-*Ofa*-UpF | *ofa* | Up | **ggaattgtgagcggataacaatttcacacaggaaacagctg**gggcatcagtaccttgcact |
| CMR5c-*Ofa*-UpR |  |  | **atacaggttgtgcagggcaa**caggacgatctccattcgtt |
| CMR5c-*Ofa*-DownF |  | Down | **aacgaatggagatcgtcctg**ttgccctgcacaacctgtat |
| CMR5c-*Ofa*-DownR |  |  | **ccaggcaaattctgttttatcagaccgcttctgcgttctgat**gttcgatcaacggcaacagt |
| CMR5c-*LuxRup*-UpF | *luxRup* | Up | **ggaattgtgagcggataacaatttcacacaggaaacagctg**atctgcccttgaatctgcac |
| CMR5c-*LuxRup*-UpR |  |  | **ggtaggtctcgatggagctg**aattcggtgtttccaggttg |
| CMR5c-*LuxRup*-DownF |  | Down | **caacctggaaacaccgaatt**cagctccatcgagacctacc |
| CMR5c-*LuxRup*-DownR |  |  | **ccaggcaaattctgttttatcagaccgcttctgcgttctgat**gatgaccctgatgagcctgt |
| CMR5c-*luxRdown*-upF | *luxRdown* | Up | **ggaattgtgagcggataacaatttcacacaggaaacagctg**agcgtgaagatcaaggacgg |
| CMR5c-*luxRdown*-upR |  |  | **ccacccatatcacccagctg**aaccgcgagatggaagtctg |
| CMR5c-*luxRdown*-downF |  | Down | **cagacttccatctcgcggtt**cagctgggtgatatgggtgg |
| CMR5c-*luxRdown*-downR |  |  | **ccaggcaaattctgttttatcagaccgcttctgcgttctgat**gggcgtctctggtctttctc |

^a^ Primer extensions are shown in bold font

**Supplementary Table** **2** Mass spectrometry data (UPLC-MS and LC-MS) of orfamide derivatives isolated and purified from plant-associated fluorescent pseudomonads strains. Mass to charge ratio (*m*/*z*) of corresponding peaks shown in Figure 3 are listed, the slash (/) indicates mass data were not detected by LC-MS.

| **Comp.** | **UPLC-MS** | | | **LC-MS** | |
| --- | --- | --- | --- | --- | --- |
|  | **[M+H]^+^** | **[M+Na]^+^** | **[M+K]^+^** | **[M+H]^+^** | **[M+2H]^2+^** |
| A | 1295.7 | 1317.6 | 1333.7 | / | / |
| B | 1281.8 | 1303.8 | 1319.6 | 1281.6 | 641.4 |
| C | 1267.7 | 1289.6 | 1305.7 | / | / |
| D | 1253.8 | 1276.0 | 1291.9 | 1253.6 | 627.5 |
| E | 1279.8 | 1301.8 | 1317.9 | 1279.7 | 640.5 |
| F | 1307.7 | 1329.7 | 1345.8 | 1307.7 | 654.5 |
| G | 1309.6 | 1331.9 | 1347.7 | 1309.7 | 655.5 |

**Supplementary Table** **3** NMR data (^1^H and ^13^C chemical shift *δ*, ppm; HNHα scalar coupling, *J*, Hz) of orfamide B, orfamide F and orfamide G isolated and characterized from supernatant of *Pseudomonas* sp. CMR5c ^a^

| **Residue** | **Position** | **Orfamide B** | | **Orfamide F** | | **Orfamide G** | |
| --- | --- | --- | --- | --- | --- | --- | --- |
|  |  | ***δ*_H_; (*J*, Hz)** | ***δ*_C_** | ***δ*_H_; (*J*, Hz)** | ***δ*_C_** | ***δ*_H_; (*J*, Hz** | ***δ*_C_** |
| Leu1 | NH | 7.70m |  | n.a. |  | 7.76m |  |
|  | CHα | 3.88m | 53.6 | n.a. | n.a. | 3.89m | 53.6 |
|  | CO |  | n.a. |  |  |  |  |
|  | CH_2_β | 1.62m;1.69m | 39.3 | 1.67m; 1.70m | 39.3 | 1.63m; 1.71m | 39.3 |
|  | CHγ | 1.66m | 25.2 | 1.78m | 24.4 | 1.67m | 25.2 |
|  | CH_3_δ | 0.94m | 23.1 | 0.93m | 23.3 | 0.93m | 23.3 |
|  | CH_3_δ | 0.90m | 22.1 | 0.89m | 22.0 | 0.89m | 22.0 |
| Glu2 | NH | 7.84m |  | n.a. |  | 7.92d |  |
|  | CHα | 4.01m | 57.0 | 4.00m | 57.1 | 4.01m | 57.0 |
|  | CO |  | n.a. |  |  |  |  |
|  | CH_2_β | 2.01q (7.2) | 26.2 | 2.02m | 27.8 | 2.02m | 26.4 |
|  | CH_2_γ | 2.47m | 30.5 | n.a. | n.a. | 2.46q (6.2) | 30.7 |
|  | COδ |  | 174.6 |  | n.a. |  | n.a. |
|  | OH | n.a. |  | n.a. |  | n.a. |  |
| Thr3 | NH | 7.94d (6.6) |  | n.a. |  | 7.96d (6.8) |  |
|  | CHα | 4.10dd  (6.6; 10.7) | 61.5 | 4.25m | 61.4 | 4.13dd (11.2; 6.8) | 61.4 |
|  | CO |  | n.a. |  | n.a. |  | n.a. |
|  | CHβ | 5.20sext | 70.4 | 5.27m | 70.7 | 5.23sext | 70.3 |
|  | CH_3_γ | 1.35d (6.0) | 18.8 | 1.36m | 18.6 | 1.35d (6.0) | 18.8 |
| Val4 | NH | 7.35d (6.0) |  | 7.63m |  | 7.41d (5.9) |  |
|  | CHα | 3.58dd  (10.5; 6.0) | 64.8 | 3.51m | 64.7 | 3.57dd (5.9; 10.3) | 64.6 |
|  | CO |  | n.a. |  | n.a. |  | n.a. |
|  | CHβ | 2.23m | 30.4 | 2.21m | 30.3 | 2.23m | 30.3 |
|  | CH_3_γ | 0.93m | 20.9 | 0.96m | 20.8 | 0.94m | 20.9 |
|  | CH_3_γ | 0.92m | 19.1 | 0.91m | 19.2 | 0.92m | 19.1 |
| Leu5 | NH | 7.84d (4.0) |  | 7.84m |  | 7.85d (6.6) |  |
|  | CHα | 4.02m | 55.2 | 4.01m | 55.1 | 4.01m | 55.2 |
|  | CO |  | n.a. |  | n.a. |  | n.a. |
|  | CH_2_β | 1.58m; 1.72m | 40.2 | 1.54m; 1.74m | 40.2 | 1.57m; 1.72m | 40.2 |
|  | CHγ | 1.78m | 25.4 | 1.78m | 24.4 | 1.79m | 25.4 |
|  | CH_3_δ | 0.87d (6.6) | 23.4 | 0.87m | 23.4 | 0.87m | 23.4 |
|  | CH_3_δ | 0.89m | 20.8 | 0.87m | 21.0 | 0.88m | 20.8 |
| Ser6 | NH | 7.32d (6.3) |  | 7.27m |  | 7.31d (6.5) |  |
|  | CHα | 4.24m | 57.17 | 4.30m | 56.4 | 4.26m | 57.0 |
|  | CO |  | n.a. |  | n.a. |  | n.a. |
|  | CH_2_β | 3.94m;4.03m | 61.9 | 3.87m; 4.03m | 62.5 | 3.94m; 4.04m | 62.0 |
|  | OHγ | n.a. |  | n.a. |  | n.a. |  |
| Leu7 | NH | 7.55d (7.6) |  | 7.44m |  | 7.54d (7.5) |  |
|  | CHα | 4.34m | 54.0 | 4.30m | 53.9 | 4.34m | 54.0 |
|  | CO |  |  |  |  |  |  |
|  | CH_2_β | 1.51m; 1.66m | 42.4 | 1.47m; 1.66m | 42.0 | 1.51m; 1.66m | 42.4 |
|  | CHγ | 1.73m | 25.2 | 1.78m | 24.4 | 1.73m | 25.2 |
|  | CH_3_δ | 0.94m | 23.2 | 0.91m | 21.4 | 0.94m | 23.2 |
|  | CH_3_δ | 0.90m | 21.6 | 0.95m | 23.4 | 0.90m | 21.6 |
| Leu8 | NH | 6.94d (6.9) |  | 7.21m |  | 6.93 (*6.5*) |  |
|  | CHα | 4.28m | 53.9 | 4.26m | 54.1 | 4.28m | 54.0 |
|  | CO |  | n.a. |  | n.a. |  | n.a. |
|  | CH_2_β | 1.67m; 1.80m | 42.1 | 1.65m; 1.76m | 41.1 | 1.67m; 1.79m | 41.9 |
|  | CHγ | 1.85m | 25.6 | 1.78m | 24.4 | 1.85m | 25.6 |
|  | CH_3_δ | 0.96d (6.5) | 23.6 | 0.96m | 23.3 | 0.96d (6.5) | 23.5 |
|  | CH_3_δ | 0.88m | 21.1 | 0.88m | 21.6 | 0.88m | 21.2 |
| Ser9 | NH | 7.03d (4.2) |  | 7.47m |  | 7.09m |  |
|  | CHα | 4.05m | 58.9 | 4.13m | 58.3 | 4.07m | 58.7 |
|  | CO |  | n.a. |  | n.a. |  | n.a. |
|  | CH_2_β | 3.74dd (11.2; 3.4); 3.88m | 62.0 | 3.73m; 3.84m | 62.6 | 3.75dd (11.7; 3.9); 3.87m | 62.6 |
|  | OHγ | n.a. |  | n.a. |  | n.a. |  |
| Val10 | NH | 6.46d (9.8) |  | 6.66m |  | 6.49d (9.2) |  |
|  | CHα | 4.54dd (9.8; 3.8) | 57.1 | 4.41m | 58.2 | 4.53m | 57.2 |
|  | CO |  | n.a. |  | n.a. |  | n.a. |
|  | CHβ | 2.19m | 31.3 | 2.04m | 31.6 | 2.17m | 31.3 |
|  | CH_3_γ | 0.83d (6.9) | 19.2 | 0.84m | 19.5 | 0.83d (6.9) | 19.2 |
|  | CH_3_γ | 0.69d (6.7) | 17.6 | 0.73m | 18.2 | 0.69d (6.7) | 17.6 |
| Fatty acid | CO 1' |  |  |  |  |  |  |
|  | CH_2_ 2' | 2.32dd 14.3; 9.4); 2.40dd (14.3; 4.0) | 44.4 | n.a. | n.a. | 2.40dd (14.2; 3.9); 2.32dd (14.1; 9.2) | 44.4 |
|  | CH 3' | 3.99m | 69.6 | 4.42m | 68.7 | 3.99m | 69.6 |
|  | OH 3' | n.a. |  | n.a. |  | n.a. |  |
|  | CH_2_ 4' | 1.47m | 38.1 | n.a. ^b^ | n.a. ^b^ | 1.48m | 38.1 |
|  | CH_2_ 5' | 1.28m; 1.40m | 26.3 | n.a. ^b^ | n.a. ^b^ | 1.31m;1.42m | 26.3 |
|  | CH_2_ 6' | n.a. | n.a. | n.a. ^b^ | n.a. ^b^ | 1.27m | 30.2 |
|  | CH_2_ 7' | n.a. | n.a. | n.a. ^b^ | n.a. ^b^ | 1.27m | 30.2 |
|  | CH_2_ 8' | n.a. | n.a. | n.a. ^b^ | n.a. ^b^ | n.a. | n.a. |
|  | CH_2_ 9' | n.a. | n.a. | n.a. ^b^ | n.a. ^b^ | n.a. | n.a. |
|  | CH_2_ 10' | n.a. | n.a. | n.a. ^b^ | n.a. ^b^ | n.a. | n.a. |
|  | CH_2_ 11' | n.a. | n.a. | n.a. ^b^ | n.a. ^b^ | n.a. | n.a. |
|  | CH_2_ 12' | n.a. | n.a. | n.a. ^b^ | n.a. ^b^ | n.a. | n.a. |
|  | CH_2_ 13' | 1.28m | 23.4 | n.a. ^b^ | n.a. ^b^ | n.a. | n.a. |
|  | CH_3/2_ 14' | 0.89m | 14.3 | n.a. ^b^ | n.a. ^b^ | n.a. | n.a. |
|  | CH_2_ 15' |  |  | 1.29m | 23.3 | 1.30m | 23.3 |
|  | CH_3_ 16' |  |  | 0.88m | 14.3 | 0.88m | 14.3 |

^a^ The NMR spectra of corresponding compounds were recorded in CD_3_CN solution at 298 K.

n. a. indicates the chemical shifts that were not assigned

^b^ Two mutually unresolved resonances, at ^1^H and ^13^C chemical shifts of 5.35 ppm and 130.6 ppm respectively, are attributed to a double bond at undetermined position in the fatty acid moiety. These correlate to a neighboring CH_2_ signal at 2.02 ppm and 27.8 ppm ^1^H and ^13^C chemical shifts respectively. Using a similar reasoning used for the CLP xantholysin C in the supporting information of Li et al (2013), the value of the latter ^13^C chemical shift is indicative for a *cis* configuration of the double bond. Using the ^13^C chemical shift prediction in the ChemDraw Ultra 15 software (*PerkinElmer, Inc.*), the chemical shifts of both CH_2_ units neighboring an isolated double bond in a linear fatty acid chain is indeed predicted as 27.7 ppm for a *cis* configuration and 33.7 ppm for a *trans* configuration

**Reference**

Li, W., Rokni-Zadeh, H., De Vleeschouwer, M., Ghequire, M. G., Sinnaeve, D., Xie, G-L, et al. (2013). The antimicrobial compound xantholysin defines a new group of *Pseudomonas* cyclic lipopeptides. *PLoS* *One* 8, e62946. doi: 10.1371/journal.pone.0062946
